# Identification of therapeutic targets in ovarian cancer through active tyrosine kinase profiling

**DOI:** 10.18632/oncotarget.4996

**Published:** 2015-08-13

**Authors:** Juan Carlos Montero, Sara García-Alonso, Alberto Ocaña, Atanasio Pandiella

**Affiliations:** ^1^ Instituto de Biología Molecular y Celular del Cáncer. CSIC-Universidad de Salamanca, Spain; ^2^ Medical Oncology Unit, University Hospital of Albacete, Spain

**Keywords:** tyrosine kinases, HER2, ovarian cancer, T-DM1

## Abstract

The activation status of a set of pro-oncogenic tyrosine kinases in ovarian cancer patient samples was analyzed to define potential therapeutic targets. Frequent activation of HER family receptor tyrosine kinases, especially HER2, was observed. Studies in ovarian cancer cell lines confirmed the activation of HER2. Moreover, knockdown of HER2 caused a strong inhibition of their proliferation. Analyses of the action of agents that target HER2 indicated that the antibody drug conjugate trastuzumab-emtansine (T-DM1) caused a substantial antitumoral effect *in vivo* and *in vitro*, and potentiated the action of drugs used in the therapy of ovarian cancer. T-DM1 provoked cell cycle arrest in mitosis, and caused the appearance of aberrant mitotic spindles in cells treated with the drug. Biochemical experiments confirmed accumulation of the mitotic markers phospho-Histone H3 and phospho-BUBR1 in cells treated with the drug. Prolonged treatment of ovarian cancer cells with T-DM1 provoked the appearance of multinucleated cells which later led to cell death. Together, these data indicate that HER2 represents an important oncogene in ovarian cancer, and suggest that targeting this tyrosine kinase with T-DM1 may be therapeutically effective, especially in ovarian tumors with high content of HER2.

## INTRODUCTION

Ovarian cancer represents the leading cause of death from gynecological malignancies in western countries [1]. The yearly estimated incidence is of 12 new cases for every 100,000 women and a death rate of 8 women for every 100,000 women. Although advances in surgical and chemotherapeutic treatments have resulted in improvements in outcome, metastatic ovarian cancer is still incurable mainly due to the lack of effective therapeutic strategies [1, 2]. Because of this, intense efforts are being carried out to define potential molecular targets that may augment survival of patients with ovarian cancer. In this respect, several reports have identified potential therapeutic targets and the efficacy of treatments against such targets is being analyzed in clinical trials in various phases of development [1]. Among such experimental treatments, agents acting on tyrosine kinases have received special attention. Thus, agents targeting vascular endothelial growth factor (VEGF) signaling have shown efficacy in preclinical models when combined to chemotherapy [3, 4]. Moreover, based on positive results obtained in clinical trials using bevacizumab-based combinations [5, 6], on November 14, 2014, the Food and Drug Administration (FDA) approved bevacizumab in combination with chemotherapy for the treatment of patients with platinum-resistant recurrent epithelial ovarian cancer. The platelet-derived growth factor receptors (PDGFR) and their ligands [7], and c-Kit [8] are overexpressed in ovarian cancers. However, Phase II trials have reported negative results in patients treated with imatinib, which targets both PDGFR and c-Kit [1].

Several reports indicate that the ErbB/HER family of receptor tyrosine kinases may represent alternative targets in ovarian cancer [1]. In fact, the epidermal growth factor receptor (EGFR) is expressed in 70% of ovarian tumors [9] and agents that target this receptor have shown antitumor activity in preclinical models [10]. However, clinical results using EGFR-targeted agents have indicated that such agents offer limited benefit [1]. Expression of the related ErbB2/HER2 transmembrane tyrosine kinase has been reported in ovarian cancer [11]. The success of therapies against this receptor in other tumoral pathologies, especially in breast cancer [12], led to the development of clinical studies aimed at the evaluation of the efficacy of such therapies in HER2 positive ovarian tumors. A phase II trial on the efficacy of trastuzumab, an antibody against HER2, in ovarian cancer patients indicated that 7% of treated patients responded to this agent, with 39% of the patients showing stabilization of the disease [13]. Pertuzumab, another anti-HER2 antibody which acts by preventing dimerization of HER2 with other HER family receptors, has been reported to augment the antitumoral properties of gemcitabine [14]. Interestingly, preclinical studies have shown that combination of trastuzumab and pertuzumab may be more efficacious than treatment with single antibodies [15]. In an RNAi-based study, Sheng and colleagues identified another HER family receptor, HER3, as a potential target in ovarian cancer [16]. RNAi against this receptor reduced ovarian cancer cell growth *in vitro* and an antibody against HER3 reduced tumor growth in mice xenografted with human ovarian cancer cells. More recently, HER3 has been reported to be a relevant player implicated in the facilitation of hematogenous spread of ovarian cancer [17].

Because of the relevance of tyrosine kinases in cancer, a study to evaluate the expression of several of these kinases in ovarian cancer was carried out. From the whole tyrosine kinase kinome, and with the purpose of identifying targets amenable for therapeutic intervention, we selected 22 tyrosine kinases against which drugs are already approved for clinical use or in advanced stages of clinical development. Using patient-derived tumoral samples, we observed that HER2 was active in most of the samples analyzed. We show that a trastuzumab-drug conjugate outweighed the antitumoral efficacy of other drugs currently used for the targeting of HER2, including trastuzumab or pertuzumab, or the small molecule HER2 inhibitor lapatinib.

## RESULTS

### Active tyrosine kinase profiling in ovarian cancer

Given the relevance of tyrosine kinases in cancer initiation/progression, we designed a study to evaluate the activation status of several tyrosine kinases in ovarian cancer. We decided to study activation status rather than expression levels, since a more accurate evaluation of their potential relevance in cancer initiation/progression is the analysis of their functionality, which is linked to their activation status [18]. The latter can be analyzed by direct activity measurements, but is more often assessed by evaluation of tyrosine phosphorylation of residues which are used as readouts of the activation status of the tyrosine kinase. Another prerequisite of the study design was to analyze tyrosine kinases against which drugs were already approved or under clinical evaluation, with the aim of rapidly translating the observed findings to the clinical setting. This restricted the analyses to 22 tyrosine kinases including receptor and cytosolic tyrosine kinases. The list of the kinases selected and drugs that act on them is shown in Table 1. Another three receptor tyrosine kinases, HER4, Dtk and EphA2, were also added to the study. The kinase HER4 was included because of the relevant role of HER receptors in cancer, even though agents against this kinase are not under clinical development. Two other kinases, Dtk and EphA2, were also included in the array. They were expected to be inactive and act as negative controls.

**Table 1:** Tyrosine kinases analyzed and available drugs that act on them

Name	Drugs
EGFR	Gefitinib, Erlotinib Cetuximab, Lapatinib, Afatinib, Neratinib
HER2	Trastuzumab, Lapatinib, Pertuzumab, T-DM1, Afatinib, Neratinib
HER3	Sapitinib, MM-121, Patritumab, Canertinib
c-Kit	Imatinib, Nilotinib
Met	Crizotinib, Cabozantinib, Foretinib
PDGFRα	Imatinib, Ponatinib Sorafenib, Pazopanib
PDGFRβ	Imatinib, Sunitinib, Axitinib Sorafenib, Pazopanib
Flt3	Quizartinib, Sunitinib, Lestaurtinib
CSF-1R	Lucitanib
Ret1	Cabozantinib, Vandetanib, Regorafenib, Ponatinib, Sunitinib
Ret2	Cabozantinib, Vandetanib, Regorafenib, Ponatinib, Sunitinib
IGF1R	Figitumumab, Ganitumab, Cixutumumab, Dalotuzumab, Robatumumab
VGFR1	Pazopanib, Sunitinib, Vatalanib
VGFR2	Ramucirumab, Pazopanib, Sunitinib, Vandetanib, Regorafenib, Lenvatinib
VGFR3	Pazopanib, Lenvatinib, Cediranib
FAK	Defactinib, GSK-2256098, PF-562,27, VS-4718
Src	Dasatinib, Saracatinib, Bosutinib
Abl	Imatinib, Nilotinib, Dasatinib, Ponatinib
FGFR1	Dovitinib, Lucitanib, BGJ398, AZD4547
FGFR2	Dovitinib, Lucitanib, BGJ398, AZD4547
FGFR3	Dovitinib, BGJ398, AZD4547
FGFR4	BGJ398, AZD4547

**Figure 1:**
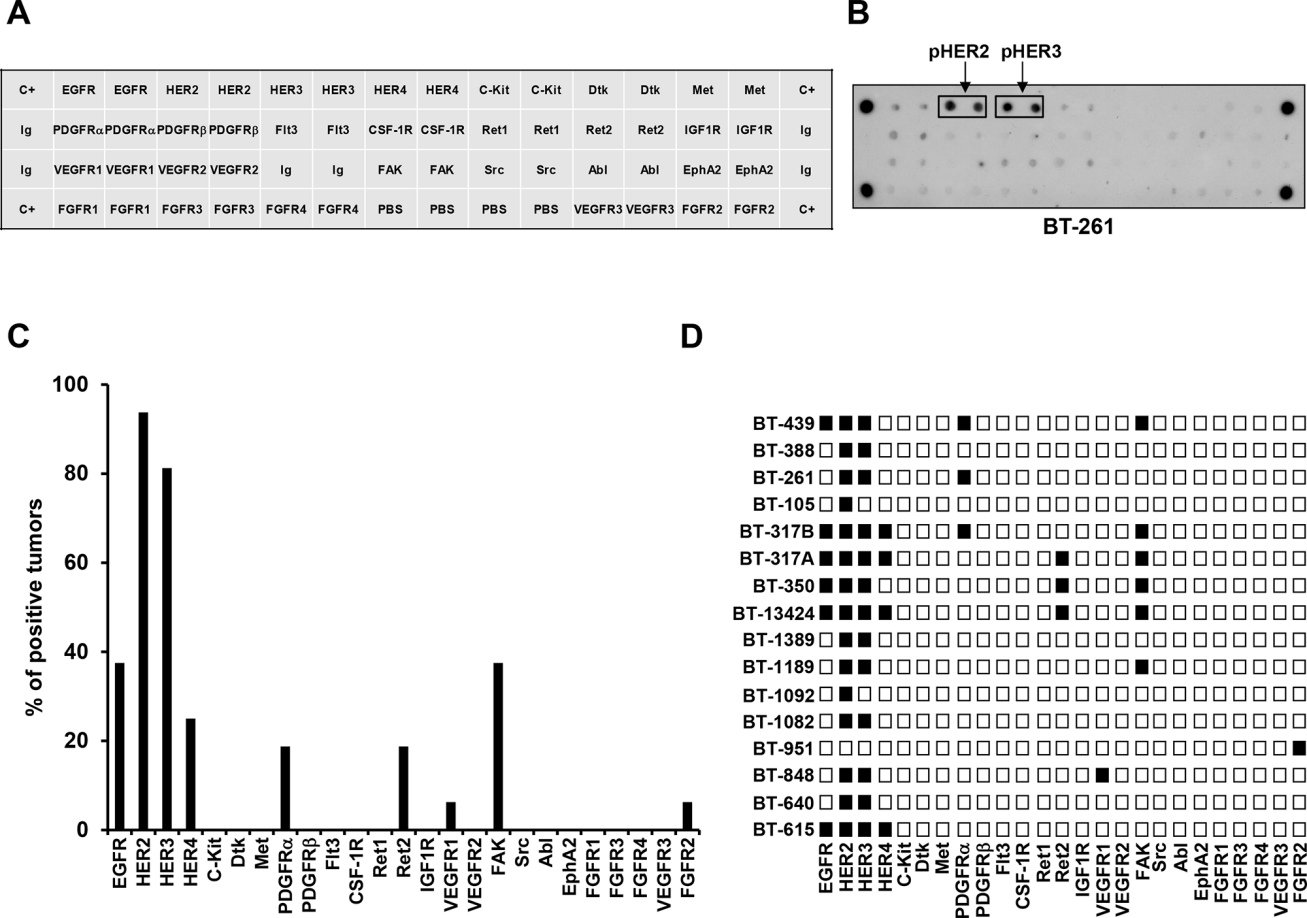
Expression of activated forms of different tyrosine kinases (TKs) in tumor samples from patients with ovarian cancer **A.** 2D map detailing the positions of the different capture antibodies used. C+: positive controls. Ig or PBS: negative controls. **B.** Image from a representative array of an ovarian tumor sample. **C.** Frequency of activation of the different phospho-TKs analyzed in the array in the ovarian tumor samples. **D.** Phospho-TKs activated in each tumor are shown by black squares.

Sixteen ovarian cancer tumors, whose patient characteristics are shown in Table 2, were analyzed by antibody arrays. Because of the lack of available commercial arrays covering all the desired tyrosine kinases we prepared the antibody arrays in house, after carefully testing different antibodies against each antigen to select the capture antibodies to be used in the blots. Duplicate spots of each antibody were adsorbed onto a nitrocellulose membrane, and protein lysates from the different ovarian tumor samples hybridized to the antibody arrays. Figure 1A describes a map with the position of the different capture antibodies in the array and Figure 1B shows data representative from an array obtained upon incubation of an ovarian tumor protein lysate. Figure 1C shows that the most frequently activated tyrosine kinases in ovarian cancer samples were HER2 (95% of patients), HER3 (80%), EGFR (37%), FAK (37%) and HER4 (25%). Of note, these analyses also revealed the frequent coexistence of several activated kinases in a single tumor (Figure 1D).

**Table 2:** Patient characteristics

**Age**	Mean: 60.37 (43–77)	%	*n* = 16
**Tumor type**	Cystoadenocarcinoma	68.75%	*n* = 11
	Tumor borderline	6.25%	*n* = 1
	Serous carcinoma	18.75%	*n* = 3
	Undifferentiated	6.25%	*n* = 1
**Stage**	IA	6.25%	*n* = 1
	IIIA	12.50%	*n* = 2
	IIB	12.50%	*n* = 2
	IIC	12.50%	*n* = 2
	IIIC	56.25%	*n* = 9
**ECOG**	0	7.70%	*n* = 1
	1	61.50%	*n* = 8
	2	23.10%	*n* = 3
	4	7.70%	*n* = 1

### HER2 knockdown restricts the proliferation of ovarian cancer cells

**Figure 2:**
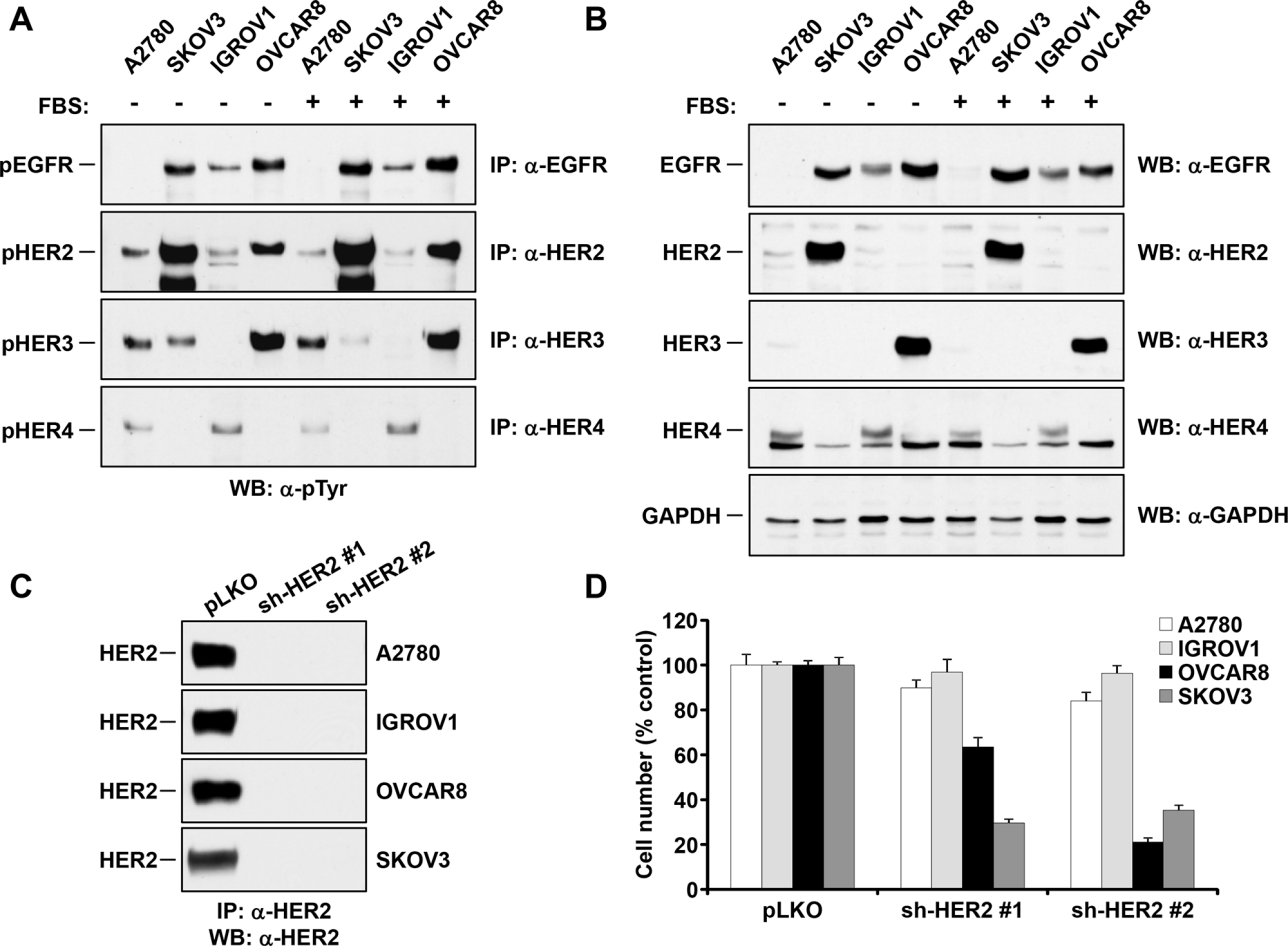
Relevance of HER2 in the proliferation of ovarian cancer cells Activation **A.** and total levels **B.** of EGFR, HER2, HER3 and HER4 in four ovarian cancer cell lines cultured for 12 hours in the presence or absence of FBS. Activated forms of the HER receptors were analyzed by immunoprecipitation with anti-receptor antibodies, followed by anti-PY blot (A). The levels of the receptors were analyzed in total cell lysates with the respective anti-receptor antibodies (B) GAPDH was used as a loading control. **C.** Knockdown of HER2 in ovarian cell lines. Cells were infected with control vector (pLKO) and viruses including two different short hairpin sequences targeting HER2. Cell extracts were obtained and the receptor expression was measured by immunoprecipitation followed by Western blot with the anti-HER2 antibody. **D.** HER2 knockdown effect on the proliferation of ovarian cancer cells. Cells were infected with the indicated shRNAs. After selection, cells were then plated and counted after 5 days. Results are plotted as the mean ± s.d. of triplicates with respect to the proliferation of untreated cultures.

The above studies indicated that HER receptors, particularly HER2, were constitutively active in a large proportion of ovarian cancer samples. To investigate the relevance of these receptors in ovarian cancer proliferation, we used four ovarian carcinoma cell lines on which the activation status of HER receptors was analyzed by immunoblotting. The cell lines SKOV3 and OVCAR8 expressed active forms of EGFR, HER2 and HER3 (Figure 2A), and expression of such activated forms was not substantially affected by the presence of serum in the media. IGROV1 cells showed expression and activation of EGFR, HER2 and HER4 receptors. The cell lines A2780 and IGROV1 expressed very small amounts of active HER2 as compared to the other cell lines (Figure 2A). A2780 did not express detectable levels of active EGFR. Of note, HER2 was the only HER family receptor expressed and activated in all four ovarian cancer cell lines (Figure 2A and 2B). This last observation falls in line with the data obtained in patient samples using the antibody arrays, which indicated HER2 as the most frequently activated kinase.

To explore the relevance of HER2 in controlling the proliferation of ovarian cancer cells, we knocked down HER2 levels in the four ovarian cancer cell lines by the use of RNAi. From the five RNAi vectors analyzed, the two of them which caused a more important decrease in HER2 were used to carry out the functional studies. As shown in Figure 2C, knockdown of HER2 was efficiently obtained in the four cell lines by either of the two shRNAs used. Knockdown of HER2 profoundly affected proliferation of SKOV3 and OVCAR8 (Figure 2D). In contrast, little effect on cell proliferation was observed by knocking down HER2 in A2780 or IGROV1 cells.

### Pharmacological targeting of HER2 in ovarian cancer

**Figure 3:**
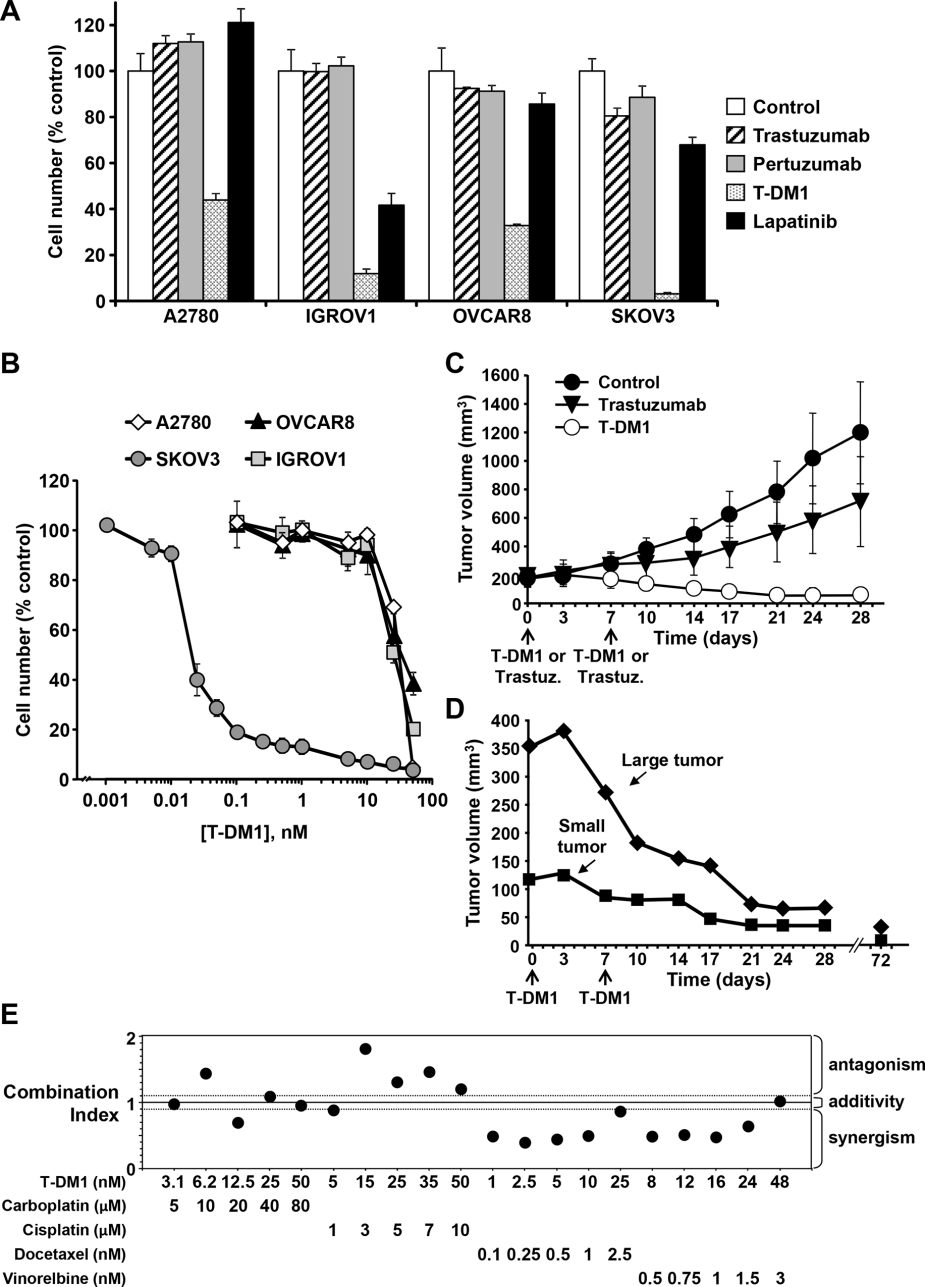
Antitumoral action of T-DM1 **A.** Effect of different drugs targeting HER2 on the proliferation of four ovarian cancer cell lines. Cells were treated with 1 μM lapatinib and 50 nM of the rest of the indicated drugs for 7 days. Cells were counted and results plotted as mean ± s.d. of triplicates with respect to the proliferation of untreated cultures. **B.** Dose-response studies of the effects of T-DM1 on the four ovarian cancer cell lines. Cells were counted and results plotted as mean ± s.d. of triplicates with respect to the proliferation of control untreated cultures. **C.** Tumor sizes of control mice (*n* = 5) and those treated with trastuzumab and T-DM1 (*n* = 5 per group) with the schedules and doses indicated under ‘Materials and methods’. Data represent the mean ± s.d. Statistical analyses were performed on the measurements obtained at the end of the experiment (day 28), with the following results: Control vs trastuzumab, *P* = 0.504; Control vs T-DM1, *P* = 0.0001; trastuzumab vs T-DM1, *P* = 0.004. *P* values were calculated using Student *t* test (two-sided). **D.** Absence of relapse for large and small tumors after regression caused by T-DM1 treatment. The data plotted correspond to the measurements of two different tumors from two different animals which were followed for up to 72 days from the start of the treatment (65 days after the last T-DM1 injection). **E.** Effect of the combination of T-DM1 with chemotherapeutic agents used in ovarian cancer (carboplatin, cisplatin, docetaxel and vinorelbine). SKOV3 cells were treated with the indicated doses of the drugs and their MTT metabolization were measured. Combination indexes for the different drug combinations were obtained using the CalcuSyn program and plotted.

The antiproliferative effects observed using the genetic targeting of HER2 in ovarian cancer cells suggested that pharmacologically acting on this receptor could exert an antitumoral action in ovarian cancer. We therefore concentrated in evaluating the effect of highly specific drugs approved for the targeting of HER2. The four ovarian cancer cell lines were treated with trastuzumab, pertuzumab, lapatinib or the antibody-drug conjugate trastuzumab-emtansine (T-DM1) [19]. Control or treated cell cultures were counted after 7 days of treatment. Addition of trastuzumab or pertuzumab did not affect the proliferation of the four ovarian cancer cell lines analyzed (Figure 3A). In contrast, a strong antiproliferative effect was observed in the case of T-DM1 in all the cell lines explored. The most sensitive cell line to T-DM1 was the SKOV3, while the less sensitive resulted to be the A2780. In SKOV3 cells lapatinib slightly reduced proliferation, while in OVCAR8 and A2780 the effect of that drug was marginal. Interestingly, lapatinib substantially inhibited the proliferation of IGROV1 cells, even though this cell line was quite insensitive to HER2 knockdown. Interestingly, these cells were also quite sensitive to the action of T-DM1. Dose response curves performed on the four ovarian cancer cell lines confirmed the high degree of sensitivity of SKOV3 cells to T-DM1 with respect to the other cell lines (Figure 3B). In fact, under these conditions, the IC_50_ value for the antiproliferative effect of T-DM1 on SKOV3 cells was in the low picomolar range (IC_50_=20 pM), while for the rest of the ovarian cancer cell lines the IC_50_ values were > 10 nM.

### T-DM1 is effective *in vivo* and potentiates the action of standard of care drugs

Part of the antitumoral effect of trastuzumab is due to antibody-dependent cellular toxicity immunological mechanisms [20]. Therefore, it is possible that its antitumoral action on ovarian cancer cells could be restricted in the *in vitro* conditions. Because of this, *in vivo* experiments comparing the antitumoral action of trastuzumab and T-DM1 were performed using mice injected with SKOV3 cells (Figure 3C). SKOV3 cells were selected for these studies because of its relatively high complement of HER2 receptors and their sensitivity to T-DM1 *in vitro*. These studies showed that trastuzumab slowed tumor growth *in vivo* when compared to the growth of tumors in untreated mice. However, the tumors continued to increase in size along time. In fact, no regressions of the tumors were observed in mice treated with trastuzumab. In this experimental setting T-DM1 not only prevented tumor growth but produced regression of the tumors. In fact, after two doses of T-DM1 the tumors in some mice were undetectable after 21 days from the initial injection of the drug. Moreover, no relapses were observed at the site of injection for up to 72 days after discontinuation of the treatments (Figure 3D). Biochemically, trastuzumab and T-DM1 did not affect the total level of HER2 in the tumors (Supplementary Figure 1). T-DM1 decreased the amount of pHER3 and pAKT to a higher extent than trastuzumab. Increased levels of pHER2 were observed in the tumors of mice treated with trastuzamab when compated to untreated or T-DM1-treated mice. Together, these data indicated that T-DM1 exerted an important antitumoral effect on ovarian cancer cells which clearly outweighd the effect of trastuzumab.

Most antitumoral treatments are based on drug combinations which have clearly demonstrated superior antitumoral activities as compared to single treatments. Because of this, whether T-DM1 potentiated the action of chemotherapeutical drugs used in the ovarian cancer clinic was investigated. To this end, combinations of T-DM1 with cisplatin, carboplatin, docetaxel or vinorelbine were tested in the SKOV3 and A2780 cells and the results analyzed by the Chou-Talalay algorithm [21]. Synergistic effects of T-DM1 with docetaxel and vinorelbine were observed (Figure 3E and Supplementary Figure 2).

### T-DM1 arrests ovarian cancer cells in mitosis

**Figure 4:**
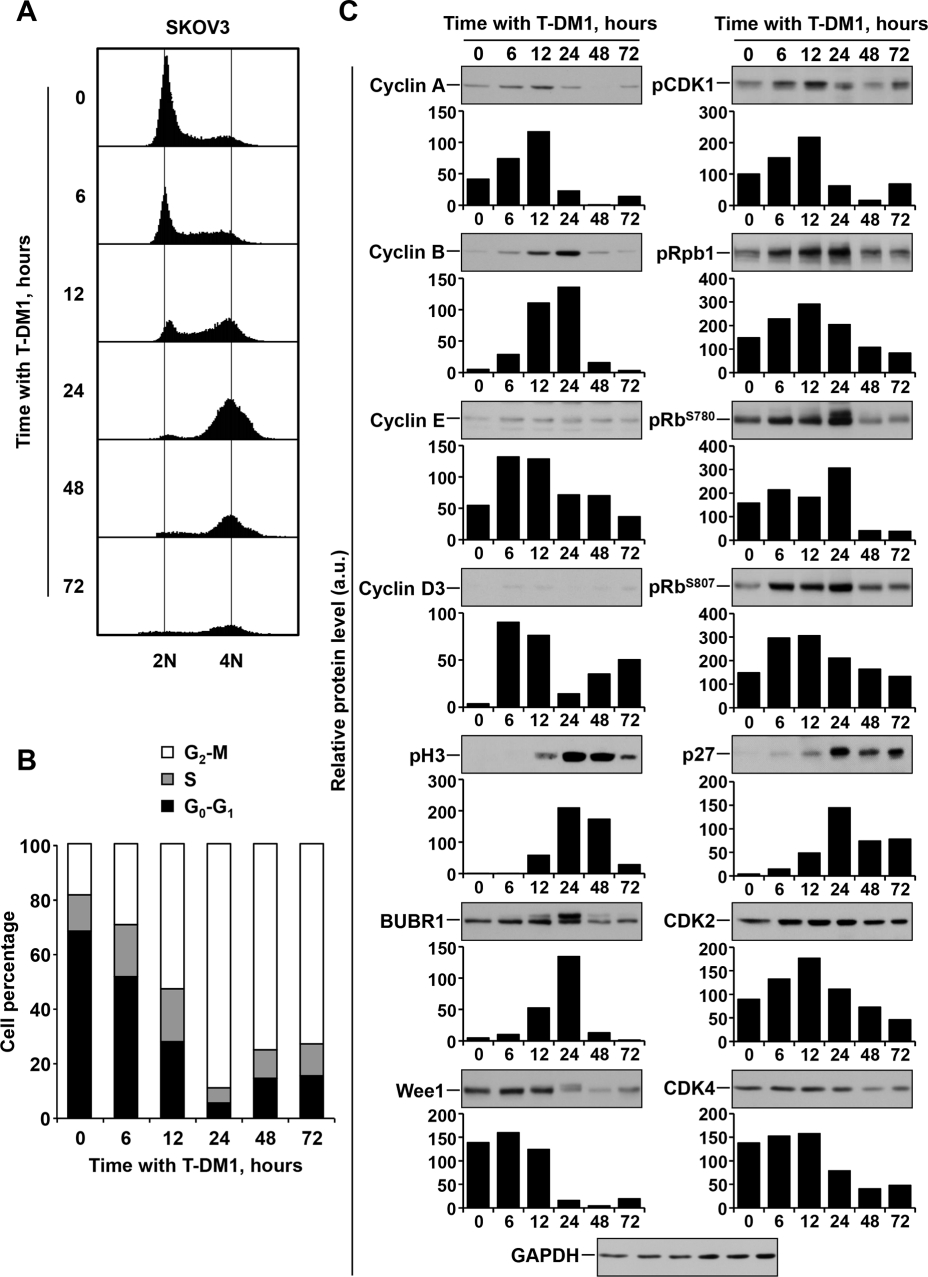
Effect of T-DM1 on the cell cycle of SKOV3 cells Cells were treated with 50 nM T-DM1 for the indicated times and cell-cycle phases were quantitatively analyzed by PI staining and FACS. **A.** Cell cycle profile. **B.** Histograms representing the percentage of cells in the different phases of the cell cycle. **C.** SKOV3 cells were treated with T-DM1 (50 nM) and lysed at the indicated times. The expression of different proteins was determined by Western blot. GAPDH was used as loading controls. Bar graph quantitative analysis of the intensity of the bands with respect to GAPDH is shown.

The decrease in cell number caused by T-DM1 could be due to inhibition of proliferation, stimulation of cell death or both. To investigate the mechanism of the antiproliferative effect of T-DM1 on ovarian cancer, SKOV3 cells were stained with propidium iodide and analyzed cytometrically at different times after addition of the drug. Treatment with T-DM1 caused accumulation of the cells in the G2/M phase of the cell cycle (Figure 4A and 4B). This effect was clear at 24 hours of treatment. At later times, although also evident, such effect was more difficult to appreciate due to the action of the drug on the cell cycle profiles.

To study whether T-DM1 arrested cells in G2 or M in SKOV3 cells, biochemical analyses of proteins whose levels mark different cell cycle phases were performed. To this end, SKOV3 cells were treated with T-DM1 for different times and cell lysates prepared to analyze the status of those marker proteins. Treatment with T-DM1 caused accumulation of the mitotic markers pHistone H3 and phosphorylated BUBR1, reaching a peak at 24 hours of treatment (Figure 4C). In addition, at 24 hours of treatment with T-DM1 dephosphorylation of CDK1 and a decrease in wee1, both events required for entry of cells in mitosis, were detected [22]. In addition, an increase of cyclin B, which acts partnering with CDK1 to allow entry of cells in mitosis and progression along prophase and metaphase, was also observed. Interestingly, an increase in the levels of p27 was also observed.

### T-DM1 causes mitotic catastrophe of ovarian cancer cells

**Figure 5:**
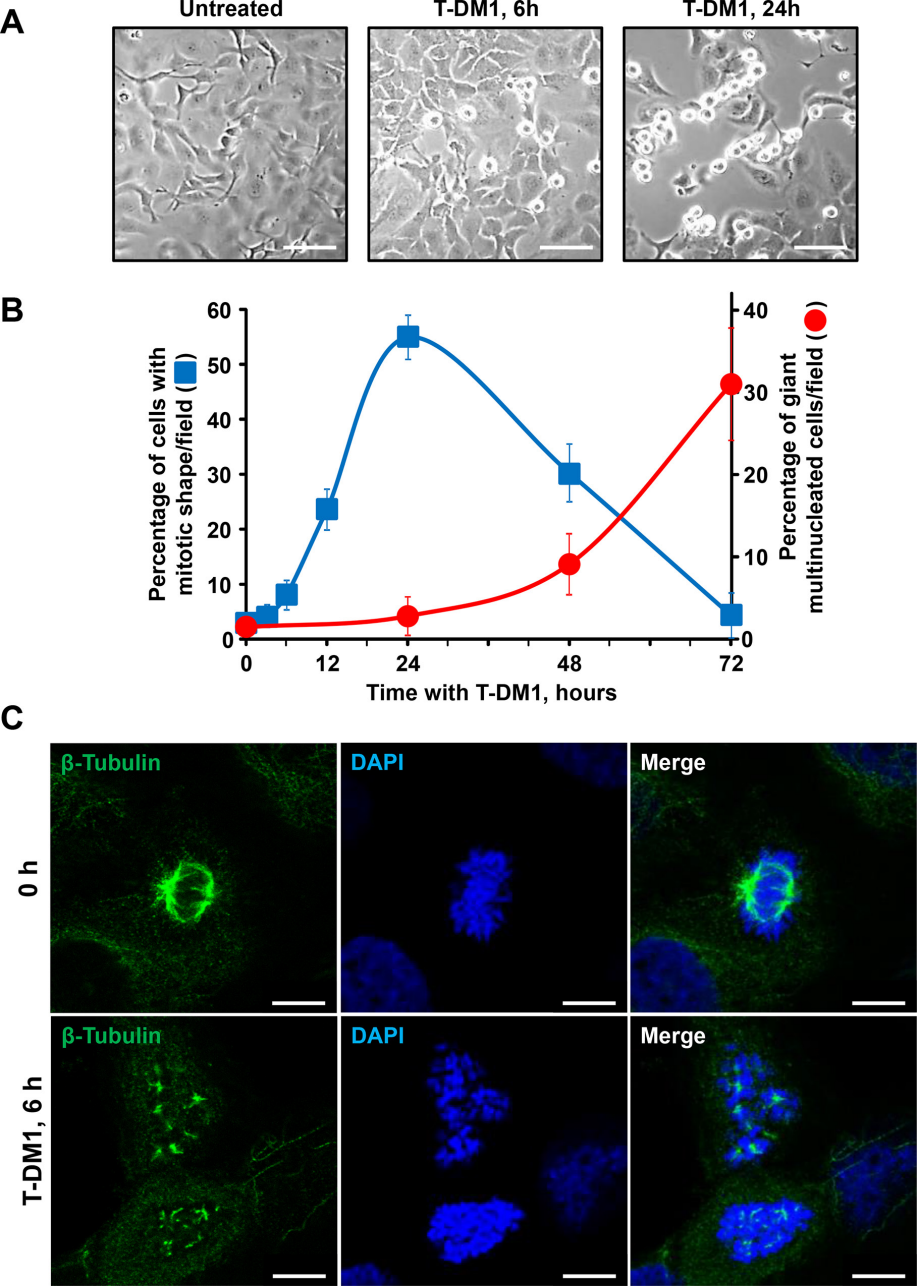
Mitotic arrest caused by T-DM1 in SKOV3 cells **A.** Effect of T-DM1 on the morphology of SKOV3 cells grown as monolayers. 1.5 × 10^5^ cells were plated in 35-mm dishes, allowed to adhere for 24 hours, then T-DM1 (50 nM) was added. The images were taken at the indicated points at x10 magnification. Scale bar = 40 μm. **B.** Percentage of cells with mitotic shape and giant multinucleated cells (GMC) in SKOV3 monolayers. Cells were treated with 50 nM T-DM1 and stained with DAPI and β-tubulin at the indicated points. GMC were defined as cells with at least three nuclei. Cells with mitotic shape and GMC were counted on a minimum of 10 randomly selected fields at x63 magnification. Data represent the average of positive cells (percentage) ± s.d./field. **C.** Effect of T-DM1 on spindle assembly and organization. SKOV3 were seeded on coverslips and treated with T-DM1 (50 nM) for 6 hours. Cells were fixed and stained for β-tubulin (green) and DNA (blue). Scale bar = 7,5 μm.

Morphologically, T-DM1 caused progressive cell rounding in cultures of SKOV3 cells, consistent with accumulation of cells in mitosis (Figure 5A and 5B). This accumulation of cells with a mitotic shape reached a peak at 24 hours decreasing thereafter (Figure 5B). Immunofluorescence analyses showed that T-DM1 caused appearance of aberrant mitotic spindles as soon as 6 hours after addition of the drug (Figure 5C).

**Figure 6:**
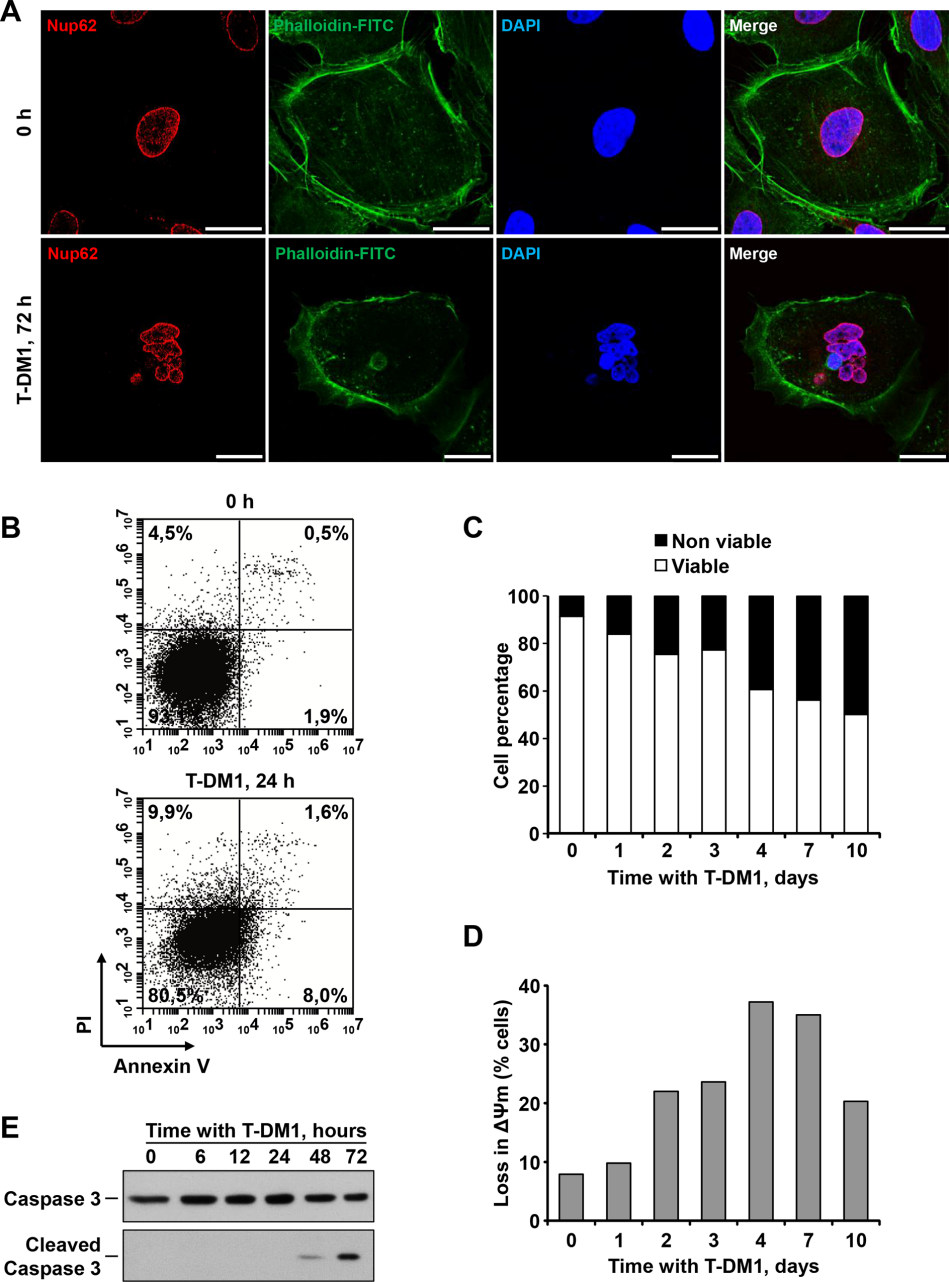
T-DM1 provokes multinucleation and mitotic catastrophe in SKOV3 cells **A.** Detection of giant multinucleated cells after T-DM1 treatment. SKOV3 were seeded on coverslips and treated with 50 nM T-DM1 for 72 hours. Cells were fixed and stained for nucleoporin p62 (red), actin (green) and DNA (blue). Scale bar = 25 μm. **B.** Effect of T-DM1 on Annexin V/PI staning of cells treated for 24 hours with the drug. SKOV3 were treated with 50 nM for the indicated times, double stained with Annexin V-FITC/PI and analyzed by flow cytometry. **C.** Effect of T-DM1 on cell viability. The bar graph represents the percentage of viable (Annexin V-negative/PI-negative) and non-viable cells. **D.** Effect of T-DM1 on the mitochondrial membrane potential of SKOV3 cells. Cells were cultured and treated as above, harvested and stained to assess their membrane potential as described under the Materials and Methods section. **E.** Action of T-DM1 on caspase 3. SKOV3 were treated with 50 nM T-DM1, lysed at indicated points and immunoblot analysis was done for caspase-3 or cleaved caspase-3.

Immunofluorescence analyses also demonstrated the appearance of giant multinucleated cells at longer incubation times (48 and 72 hours) with T-DM1 (Figure 5B and 6A). The rise in the appearance of these multinucleated cells followed an inverse time-course with respect to the mitotic cells, i.e. the progressive decrease in mitotic cells after 24 hours was followed by a concomitant progressive appearance of multinucleated cells (Figure 5B).

The presence of these multinucleated cells, likely caused by deficient spindle assembly due to the maytansine derivative, was indicative of mitotic catastrophe, a form of cell death triggered by deficient mitotic progression [23, 24]. Annexin V/propidium iodide staining experiments indicated that T-DM1 was unable to substantially increase apoptotic cell death at early (<24 hours) incubation times (Figure 6B and 6C). However, extension of the incubation with the drug for up to 10 days provoked a progressive increase in cell death (Figure 6C).

Treatment with T-DM1 caused a progressive derangement in the mitochondrial membrane potential (Figure 6D), indicative of increased permeability of the mitochondrial outer membrane, a process which is linked to cell death [25]. Increases in mitochondrial outer membrane permeability allow the release of cell death mediators from the mitochondrial intermembrane space, which may cause activation of executioner caspases, important mediators of cell death processes [25]. Treatment of SKOV3 cells with T-DM1 caused increase in cleaved caspase 3, which represents the active form of this caspase (Figure 6E). These increases in cleaved caspase 3 were observed at 48 and 72 hours of treatment with the drug, times at which loss in mitochondrial membrane potential was already evident (Figure 6D). Together, these data indicate that treatment of SKOV3 cells with T-DM1 caused cell cycle arrest which is temporally followed by progression into cell death.

## DISCUSSION

The dismal prognosis of ovarian cancer in the metastatic setting has driven much research with the aim of developing therapeutic strategies to improve disease outcome [2]. Given the relevance of tyrosine kinases in cancer initiation/progression and the benefits of their targeting in other oncological diseases [26], we evaluated the activation status of several tyrosine kinases in tumor samples surgically resected from patients with ovarian cancer.

Antibody array analyses of the ovarian tumoral samples indicated that a large proportion of these tumors expressed activated forms of HER receptors, particularly HER2. Overexpression of HER2 has been reported in <10% of ovarian tumors [1], while in our active kinase arrays the level of HER2 tyrosine phosphorylation was much higher (>90%). This observation indicated that the number of tumors in which HER2 was active may be higher than expected from the expression studies. Moreover, if HER2 is important in the pathophysiology of these tumors, then a substantial proportion of ovarian cancer patients could benefit from the targeting of HER2. Because of this, studies aimed at defining the biological relevance of HER2 in ovarian cancer were performed using model cell lines bearing different complements of HER2 receptors. In all the cell lines analyzed, HER2 and its phosphorylated form were detected, although at different levels. Genetic studies using shRNAs targeting HER2 showed that in SKOV3 and OVCAR8 cells, knockdown of HER2 resulted in substantial growth inhibition. In contrast, in A2780 and IGROV1, HER2 knockdown did not substantially affect their proliferation. These differences in the sensitivity to HER2 knockdown suggest that certain cell lines (SKOV3 and OVCAR8) may depend on HER2 signaling for proliferation. In contrast, even though A2780 and IGROV1 cell lines expressed HER2, signaling by this receptor does not appear to significantly contribute to their proliferation. These results raise the question of why lapatinib affected proliferation of IGROV1 while HER2 knockdown did not. This effect of lapatinib on IGROV1 cells may be explained by an off-target action of the drug, as has already been reported in other systems [27].

Several relevant considerations from the therapeutic point of view have to be made with respect to the HER2 knockdown experiments. SKOV3 could be considered a model of ovarian tumors overexpressing HER2 which could be suitable for therapeutic intervention by adequately targeting this receptor. In addition to HER2 overexpression, other factors may contribute to HER2 dependency in ovarian cancer. In this respect, it is interesting that targeting the neuregulins, a set of ligands which activate HER receptors [28], impedes proliferation of ovarian carcinoma cells [16].

The *in vitro* analyses of the effect of several approved anti-HER2 agents on ovarian cancer cell lines showed limited efficacy of trastuzumab or pertuzumab, in line with a previously published *in vivo* study [15]. In contrast, the trastuzumab derivative T-DM1 showed strong antitumoral properties both *in vitro* and *in vivo* on ovarian cancer cells. Because of this, we undertook a comprehensive study to characterize the molecular response of ovarian cancer cells to T-DM1. Dose-response curves indicated that the most sensitive cell line was SKOV3. This may be explained by the concurrence of two situations: the high expression level of HER2 and the addiction to HER2 observed in SKOV3 cells. Mechanistically, T-DM1 provoked cell cycle blockade in mitosis, as indicated by the accumulation of cells in this phase of the cell cycle and the increased amounts of proteins such as pHistone H3 and pBUBR1, which mark this phase of the cell cycle. The accumulation of cells in mitosis could be caused by the maytansinoid derivative which was likely responsible for the disruption of normal spindle assembly observed in cells treated with T-DM1. This caused augmented numbers of mitotic cells in the cultures, which then evolved into multinucleated cells. While the direct effect of the antiproliferative action of T-DM1 appears to be on the cell cycle dynamics, prolonged treatment with the drug caused cell death. Such phenomenon may be secondary to the mitotic arrest of cells and multinucleation, which characteristically lead to mitotic catastrophe, which has also been reported in breast cancer [24]. Interestingly, T-DM1 synergized with docetaxel and vinorelbine. It is therefore possible that drugs altering microtubule dynamics may augment the antitumoral action of T-DM1. In addition, T-DM1 caused an increase in the levels of p27. This effect may be due to the trastuzumab part of the compound, as this antibody has been formerly shown to provoke this biochemical effect [29]. This increase in p27 has been linked to delayed transit of the cells from G1 into S. While this effect may contribute to the composite antitumoral effect of T-DM1 it is clear that the mitotic arrest effect predominates over other effects of the drug on the cell cycle.

Of note, T-DM1 was effective not only in the cell lines that were sensitive to *in vitro* knockdown of HER2, but also in cell lines resistant to such knockdown. Thus, it is possible that in cells which are not addicted to HER2, the low levels of HER2 could act favoring the entry of T-DM1 into the cell. Alternatively, an effect of T-DM1 in these cell lines caused by constitutive internalization of extracellular milieu products could also be responsible for the antiproliferative action of T-DM1. In fact, we observed internalization of T-DM1 in all the cell lines analyzed (Supplementary Figure 3). The fact that tumoral cells are characterized by a high proliferation rate may then explain the antitumoral effect of T-DM1 on these HER2 low and knockdown-resistant cells. Moreover, pharmacokinetic studies [30] have reported plasma concentrations of T-DM1 above the maximal doses tested in our study and sustained for up to 20 days. This is important, as it is possible that ovarian patients with tumors bearing low levels of HER2 may also benefit from the antitumoral properties of T-DM1.

In summary, the work herewith reported describes the activation status of several druggable tyrosine kinases in ovarian cancer. The study reports frequent activation of HER2 in the tumoral samples and shows preclinical evidence that acting on HER2 exerts an antitumoral action on cells expressing this protein. Because of the much better effect of the trastuzumab derivative T-DM1 with respect to other clinically available drugs and its potentiation of the effect on classical standard of care drugs, the preclinical data presented here call for the clinical evaluation of T-DM1, either alone or in combination, for the therapy of ovarian cancer.

## MATERIALS AND METHODS

### Reagents and antibodies

Cell culture media, puromycin, MTT and DAPI were purchased from Sigma-Aldrich (St Louis, MO, USA). Fetal bovine serum (FBS) and penicillin/streptomycin were from Invitrogen (Gaithersburg, MD, USA). Protein A-Sepharose was from GE Healthcare Life Sciences (Piscataway, NJ, USA). Carboplatin and cisplatin were from Pfizer (Madrid, Spain). Docetaxel was from Hospira UK Ltd (Warwickshire, United Kingdom). Vinorelbine was from Pierre Fabre (Barcelona, Spain). Lapatinib was from LC Laboratories (Woburn, MA, USA). Trastuzumab-emtansine (T-DM1) was purchased from a pharmacy located in Andorra. Trastuzumab was purchased from a local pharmacy and pertuzumab was obtained from Genentech (San Francisco, CA). Other generic chemicals were purchased from Sigma, Roche Biochemicals or Merck (Darmstadt, Germany).

The antibodies against Abl, CSF-1R, EphA2, FAK, FGFR1, FGFR2, FGFR3, FGFR4, Flt3, IGF-1R, Met, PDGFRα, PDGFRβ, Ret1, Ret2, Src, pY99-HRP, cyclin B, cyclin E, Wee1, p27, CDK2, CDK4 and GAPDH were purchased from Santa Cruz Biotechnology (Santa Cruz, CA, USA). The anti-pH3, anti-p(Y^15^) CDK1, anti-pRpb1, anti-pRb (S^780^) and anti-pRb (S^807/811^), pHER2 (Y^1221/1222^), pHER3 (Y^1289^), and AKT antibodies were from Cell Signaling Technologies (Beverly, MA, USA). The anti-c-Kit, anti-cyclin A, anti-cyclin D3, anti-BUBR1, anti-caspase 3, anti-cleaved caspase 3 anti-pAKT (S^473^) and anti-Nucleoporin p62 antibodies were from BD Biosciences. The anti-Dtk, anti-VEGFR1, anti-VEGFR2 and VEGFR3 antibodies were from, R&D Systems (Minneapolis, MN, USA). The anti-β-tubulin antibody was from Sigma-Aldrich. The rabbit polyclonal anti-calnexin antibody was from Stressgen Biotechnologies Corporation (British Columbia, Canada). Horseradish peroxidase conjugates of anti-rabbit and anti-mouse immunoglobulin G were from Bio-Rad Laboratories (Hercules, CA, USA). The 4D5 anti-HER2 antibody was provided by Dr. Mark X. Sliwkowski (Genentech, San Francisco, CA, USA). The Ab-3 anti-HER2 antibody used for Western blotting was from Calbiochem (La Jolla, CA, USA). The anti-EGFR, anti-HER3 and anti-HER4 antibodies have been described previously [31].

### Cell culture and infection with lentivirus

All cell lines were cultured at 37°C in a humidified atmosphere in the presence of 5% CO_2_ and 95% air. The cell lines were provided by Dr. Faustino Mollinedo (CSIC Salamanca, Salamanca, Spain), who obtained them from the American Type Culture Collection. No authentication was conducted in the author's laboratory. OVCAR8 and SKOV3 cells were grown in Dulbecco's Modified Eagle's Medium (DMEM) and A2780 and IGROV1 in RPMI-1640 medium containing a high glucose concentration (4,500 mg/L) and antibiotics (penicillin at 100 mU/mL, streptomycin at 100 μg/mL) and supplemented with 10% FBS.

Knockdown of HER2 in ovarian cells was performed by infection with lentiviral particles. The lentiviral vectors containing short hairpin RNA (shRNA) for HER2 were obtained from Thermo Scientific (Waltham, MA, USA). A minimum of 5 different shRNA sequences were tested and the two that produced higher knockdown levels were used for the proliferation experiments. Preparation of lentiviral vectors was performed as described previously [32].

### Patient samples and antibody arrays

Tyrosine phosphorylation of different tyrosine kinases was evaluated by antibody arrays in ovarian cancer samples from patients of the University Hospital of Salamanca. Fresh tissue samples from the primary tumors were embedded in OCT (Fisher Scientific) and then in isopentane, and placed at −80°C. Selection of the tumoral tissue was performed after excision of part of the tumor and staining with hematoxylin/eosin. The tumors were minced, washed with PBS and homogenized (Dispomix, L&M Biotech, Holly Springs, NC, USA) in ice-cold lysis buffer (1.5 ml/100 mg of tumour). This homogenate was centrifuged at 10,000 xg for 20 minutes at 4°C and the supernatants were transferred to new tubes.

To perform the antibody arrays, each antibody (0.1 μg) in duplicate was spotted onto a nitrocellulose membrane. The membrane was dried at room temperature for 30 minutes and then blocked for 2 hours in Tris-buffered saline with Tween (TBST) containing 5% bovine serum albumin (BSA). The membranes were then incubated with 1 mg of protein lysates overnight at 4°C. The membranes were washed in TBST three times during 10 minutes and incubated for 2 hours with the anti–phosphotyrosine-HRP antibody (1:5,000 dilution). After three washes of 10 minutes each in TBST, membranes were incubated with the ECL Plus Western Blotting Detection System (GE Lifesciences). Breast cancer cell lysates from BT474 cells were used as the positive control, as they contain high levels of resting tyrosine phosphorylation of several cellular proteins [33]. Control Ig and PBS were spotted as negative controls. Quantification of the different spots in the array was performed using the Image Studio Digits V3.1 Odyssey program (LI-COR, Lincoln, NE, USA).

### Immunoprecipitation and western blotting

Cultured cells were washed with phosphate-buffered saline (PBS) (NaCl, 137 mM; KCl, 2.7 mM; Na_2_HPO_4_, 8 mM; KH_2_PO_4,_ 1.5 mM) and lysed in ice-cold lysis buffer (20 mM Tris–HCl [pH 7.0]; NaCl, 140 mM, EDTA; 50 mM; 10% glycerol; 1% Nonidet P-40; 1 μM pepstatin; 1 μg/mL aprotinin; 1 μg/mL leupeptin; 1 mM phenylmethyl sulfonyl fluoride; 1 mM sodium orthovanadate). Lysates were centrifuged at 10,000 xg at 4°C for 10 minutes and supernatants were transferred to new tubes with the corresponding antibody and protein A-Sepharose. Immunoprecipitations were performed at 4°C for at least 2 hours. Immune complexes were recovered by a short centrifugation at 10,000 xg for 15 seconds, followed by three washes with 1 mL cold lysis buffer. Samples were then boiled in electrophoresis sample buffer and placed on SDS-PAGE gels at varying acrylamide concentrations, depending on the molecular weight of the proteins to be analyzed. After electrophoresis, the separated proteins in the gel were transferred to polyvinylidene difluoride membranes (PVDF) (Millipore Corporation, Bedford, MA, USA). Membranes were blocked in TBST (100 mM Tris [pH 7.5]; 150 mM NaCl; 0.05% Tween 20) containing 1% BSA or 5% skimmed milk for 1–3 hours and then incubated with the corresponding antibody for 2–16 hours. After washing three times with TBST during 10 minutes, membranes were incubated with HRP-conjugated anti-mouse or anti-rabbit secondary antibodies for 30 minutes. After the secondary antibody, the membranes were washed three times with TBST and the bands were visualized by using enhanced chemiluminescence [34].

### Cell proliferation, cell cycle and apoptosis assays

Cell proliferation was assessed by MTT metabolization or cell counting. For the MTT assays, cells were seeded in 24-well plates and allowed to attach overnight in DMEM or RPMI-1640 + 10% FBS. The next day medium was replaced with complete medium containing the different drugs. After treatment, cell proliferation was analyzed by an MTT-based assay as previously described [35]. For the cell counting experiments, cells were seeded in 6-well plates and cultured as above. Once the treatment finished, cells were counted using a Z1 Coulter Particle Counter (Beckman Coulter, Pasadena, CA, USA). Unless otherwise indicated, the results are presented as the mean ± standard deviation of triplicates of a representative experiment.

To determine whether the combination of T-DM1 with chemotherapeutic agents (carboplatin, cisplatin, docetaxel and vinorelbine) was synergistic, additive or antagonist, we used the CalcuSyn v2.0 software programme (Biosoft, Ferguson, MO, USA) [35]. This program allows the calculation of the combination index based on the algorithm of Chou and Talalay. Combination index values less than 1 indicate synergism, values equal to 1 indicate an additive effect, whereas values greater than 1 indicate antagonism.

For the analysis of the cell cycle profile, cells were treated with T-DM1 for the indicated period of time and subsequently collected by pooling together the nonattached and attached cells. After washing with PBS, cells were fixed and permeabilized by ice-cold 70% ethanol overnight. Cells were centrifuged, resuspended in 500 μL of PBS containing 250 μg DNase-free RNAase A (Sigma-Aldrich) and incubated at room temperature for 2 hours. Then, 2.5 μg of propidium iodide (PI; Sigma-Aldrich) were added. DNA content and cell cycle analyses were performed by using a BD Accuri C6 flow cytometer and the C6 software (BD Biosciences).

For apoptotic analysis, cells were treated with T-DM1 for the indicated times and subsequently collected by pooling together the nonattached and attached cells. Then, cells were washed with PBS and resuspended in 100 μL of binding buffer (10 mM HEPES/NaOH [pH 7.4]; 140 mM NaCl; 2.5 mM CaCl_2_) containing 5 μL of Annexin V-fluorescein isothiocyanate (FITC; BD Biosciences) and 5 μL of 50 μg/mL PI. Cells were incubated for 15 minutes in the dark. After adding another 400 μL of binding buffer, labeled cells were analyzed in a BD Accuri C6 flow cytometer. Analyses of mitochondrial membrane potential in ovarian cancer cell lines has been described [35].

### Immunofluorescence microscopy

Cells cultured on glass coverslips were washed with PBS and fixed in 2% *p*-formaldehyde for 30 minutes at room temperature, followed by a wash in PBS. Monolayers were quenched for 10 minutes with PBS with 50 nM NH_4_Cl_2_. Cells were then permeabilized for 10 minutes with PBS supplemented with 0.1% (final concentration) Triton X-100 and then blocked in PBS with 0.2% BSA for 10 minutes. Monolayers were incubated with the indicated primary antibodies in blocking solution for 1 hour at room temperature. After three washes for 10 minutes each in PBS with 0.2% BSA, coverslips were incubated with Cy3- or Alexa 488-conjugated secondary antibodies or with a 50 mg/ml FITC-labeled phalloidin (Sigma-Aldrich) solution in PBS with 0.2% BSA for 30 minutes at room temperature. After three washes for 10 minutes each in PBS with 0.2% BSA, immunolabeled cells were counterstained with DAPI to detect cell nuclei and the slides were mounted. Samples were analyzed by regular epifluorescence microscopy or by confocal immunofluorescence microscopy using a Zeiss LSM510 system.

### Xenograft studies

Mice were manipulated at the animal facility following legal guidelines. Female BALB/c *nu*/*nu* mice (7 weeks old) were obtained from Charles River Laboratories (Wilmington, MA, USA). A total of 5×10^6^ SKOV3 cells in 100 μl of DMEM and 100 μl of Matrigel (BD Biosciences) were injected subcutaneously into the right and left flank of each mouse. When tumours reached a mean volume of 200 mm^3^, animals (*n* = 15) were randomized into three groups (with equal average tumour volumes) (vehicle (PBS) *n* = 5, trastuzumab *n* = 5, and T-DM1 *n* = 5). Mice were treated twice (day 0 and day 7) at the first week with trastuzumab (15 mg/kg) and T-DM1 (15 mg/kg) intravenously. Tumour diameters were serially measured with a calliper twice per week and tumour volumes were calculated by the following formula: volume = width^2^ × length/2. Mice treated with vehicle and trastuzumab were sacrificed on day 28. Mice treated with T-DM1 were maintained to assess whether their tumors relapsed.

### Statistical analyses

Comparisons of continuous variables between two groups were performed using a two-sided Student's *t* test. Differences were considered to be statistically significant when *P* values were less than 0.05. Statistical data are presented as the mean ± s.d. All data were analyzed using the statistical software SPSS 21.0 (SPSS Inc., Chicago, IL).

## SUPPLEMENTARY FIGURES



## References

[1] Yap TA, Carden CP, Kaye SB (2009). Beyond chemotherapy: targeted therapies in ovarian cancer. Nat Rev Cancer.

[2] Banerjee S, Kaye SB (2013). New strategies in the treatment of ovarian cancer: current clinical perspectives and future potential. Clin Cancer Res.

[3] Hu L, Hofmann J, Zaloudek C, Ferrara N, Hamilton T, Jaffe RB (2002). Vascular endothelial growth factor immunoneutralization plus Paclitaxel markedly reduces tumor burden and ascites in athymic mouse model of ovarian cancer. Am J Pathol.

[4] Garofalo A, Naumova E, Manenti L, Ghilardi C, Ghisleni G, Caniatti M, Colombo T, Cherrington JM, Scanziani E, Nicoletti MI, Giavazzi R (2003). The combination of the tyrosine kinase receptor inhibitor SU6668 with paclitaxel affects ascites formation and tumor spread in ovarian carcinoma xenografts growing orthotopically. Clin Cancer Res.

[5] Burger RA, Brady MF, Bookman MA, Fleming GF, Monk BJ, Huang H, Mannel RS, Homesley HD, Fowler J, Greer BE, Boente M, Birrer MJ, Liang SX (2011). Incorporation of bevacizumab in the primary treatment of ovarian cancer. N Engl J Med.

[6] Perren TJ, Swart AM, Pfisterer J, Ledermann JA, Pujade-Lauraine E, Kristensen G, Carey MS, Beale P, Cervantes A, Kurzeder C, du Bois A, Sehouli J, Kimmig R (2011). A phase 3 trial of bevacizumab in ovarian cancer. N Engl J Med.

[7] Henriksen R, Funa K, Wilander E, Backstrom T, Ridderheim M, Oberg K (1993). Expression and prognostic significance of platelet-derived growth factor and its receptors in epithelial ovarian neoplasms. Cancer Res.

[8] Schilder RJ, Sill MW, Lee RB, Shaw TJ, Senterman MK, Klein-Szanto AJ, Miner Z, Vanderhyden BC (2008). Phase II evaluation of imatinib mesylate in the treatment of recurrent or persistent epithelial ovarian or primary peritoneal carcinoma: a Gynecologic Oncology Group Study. J Clin Oncol.

[9] Bartlett JM, Langdon SP, Simpson BJ, Stewart M, Katsaros D, Sismondi P, Love S, Scott WN, Williams AR, Lessells AM, Macleod KG, Smyth JF, Miller WR (1996). The prognostic value of epidermal growth factor receptor mRNA expression in primary ovarian cancer. Br J Cancer.

[10] Ciardiello F, Caputo R, Bianco R, Damiano V, Pomatico G, De Placido S, Bianco AR, Tortora G (2000). Antitumor effect and potentiation of cytotoxic drugs activity in human cancer cells by ZD-1839 (Iressa), an epidermal growth factor receptor-selective tyrosine kinase inhibitor. Clin Cancer Res.

[11] Lafky JM, Wilken JA, Baron AT, Maihle NJ (2008). Clinical implications of the ErbB/epidermal growth factor (EGF) receptor family and its ligands in ovarian cancer. Biochim Biophys Acta.

[12] Ocana A, Pandiella A (2013). Targeting HER receptors in cancer. Curr Pharm Des.

[13] Bookman MA, Darcy KM, Clarke-Pearson D, Boothby RA, Horowitz IR (2003). Evaluation of monoclonal humanized anti-HER2 antibody, trastuzumab, in patients with recurrent or refractory ovarian or primary peritoneal carcinoma with overexpression of HER2: a phase II trial of the Gynecologic Oncology Group. J Clin Oncol.

[14] Makhija S, Amler LC, Glenn D, Ueland FR, Gold MA, Dizon DS, Paton V, Lin CY, Januario T, Ng K, Strauss A, Kelsey S, Sliwkowski MX (2010). Clinical activity of gemcitabine plus pertuzumab in platinum-resistant ovarian cancer, fallopian tube cancer, or primary peritoneal cancer. J Clin Oncol.

[15] Sims AH, Zweemer AJ, Nagumo Y, Faratian D, Muir M, Dodds M, Um I, Kay C, Hasmann M, Harrison DJ, Langdon SP (2012). Defining the molecular response to trastuzumab, pertuzumab and combination therapy in ovarian cancer. Br J Cancer.

[16] Sheng Q, Liu X, Fleming E, Yuan K, Piao H, Chen J, Moustafa Z, Thomas RK, Greulich H, Schinzel A, Zaghlul S, Batt D, Ettenberg S (2010). An activated ErbB3/NRG1 autocrine loop supports *in vivo* proliferation in ovarian cancer cells. Cancer Cell.

[17] Pradeep S, Kim SW, Wu SY, Nishimura M, Chaluvally-Raghavan P, Miyake T, Pecot CV, Kim SJ, Choi HJ, Bischoff FZ, Mayer JA, Huang L, Nick AM (2014). Hematogenous metastasis of ovarian cancer: rethinking mode of spread. Cancer Cell.

[18] Lemmon MA, Schlessinger J (2010). Cell signaling by receptor tyrosine kinases. Cell.

[19] Lambert JM, Chari RV (2014). Ado-trastuzumab Emtansine (T-DM1): an antibody-drug conjugate (ADC) for HER2-positive breast cancer. J Med Chem.

[20] Clynes RA, Towers TL, Presta LG, Ravetch JV (2000). Inhibitory Fc receptors modulate *in vivo* cytotoxicity against tumor targets. Nat Med.

[21] Chou TC, Motzer RJ, Tong Y, Bosl GJ (1994). Computerized quantitation of synergism and antagonism of taxol, topotecan, and cisplatin against human teratocarcinoma cell growth: a rational approach to clinical protocol design. J Natl Cancer Inst.

[22] Lapenna S, Giordano A (2009). Cell cycle kinases as therapeutic targets for cancer. Nat Rev Drug Discov.

[23] Barok M, Joensuu H, Isola J (2014). Trastuzumab emtansine: mechanisms of action and drug resistance. Breast Cancer Res.

[24] Barok M, Tanner M, Koninki K, Isola J (2011). Trastuzumab-DM1 causes tumour growth inhibition by mitotic catastrophe in trastuzumab-resistant breast cancer cells *in vivo*. Breast Cancer Res.

[25] Danial NN, Korsmeyer SJ (2004). Cell death: critical control points. Cell.

[26] Ocana A, Amir E, Seruga B, Martin M, Pandiella A (2013). The evolving landscape of protein kinases in breast cancer: clinical implications. Cancer Treat Rev.

[27] Dolloff NG, Mayes PA, Hart LS, Dicker DT, Humphreys R, El-Deiry WS (2011). Off-target lapatinib activity sensitizes colon cancer cells through TRAIL death receptor up-regulation. Sci Transl Med.

[28] Montero JC, Rodriguez-Barrueco R, Ocana A, Diaz-Rodriguez E, Esparis-Ogando A, Pandiella A (2008). Neuregulins and cancer. Clin Cancer Res.

[29] Lane HA, Beuvink I, Motoyama AB, Daly JM, Neve RM, Hynes NE (2000). ErbB2 potentiates breast tumor proliferation through modulation of p27(Kip1)-Cdk2 complex formation: receptor overexpression does not determine growth dependency. Mol Cell Biol.

[30] Krop IE, Beeram M, Modi S, Jones SF, Holden SN, Yu W, Girish S, Tibbitts J, Yi JH, Sliwkowski MX, Jacobson F, Lutzker SG, Burris HA (2010). Phase I study of trastuzumab-DM1, an HER2 antibody-drug conjugate, given every 3 weeks to patients with HER2-positive metastatic breast cancer. J Clin Oncol.

[31] Sanchez-Martin M, Pandiella A (2012). Differential action of small molecule HER kinase inhibitors on receptor heterodimerization: therapeutic implications. Int J Cancer.

[32] Borges J, Pandiella A, Esparis-Ogando A (2007). Erk5 nuclear location is independent on dual phosphorylation, and favours resistance to TRAIL-induced apoptosis. Cell Signal.

[33] Seoane S, Montero JC, Ocana A, Pandiella A (2010). Effect of multikinase inhibitors on caspase-independent cell death and DNA damage in HER2-overexpressing breast cancer cells. J Natl Cancer Inst.

[34] Cabrera N, Diaz-Rodriguez E, Becker E, Martin-Zanca D, Pandiella A (1996). TrkA receptor ectodomain cleavage generates a tyrosine-phosphorylated cell-associated fragment. J Cell Biol.

[35] Montero JC, Chen X, Ocana A, Pandiella A (2012). Predominance of mTORC1 over mTORC2 in the regulation of proliferation of ovarian cancer cells: therapeutic implications. Mol Cancer Ther.

