# Supplementary material for: Identification of therapeutic targets in ovarian cancer through active tyrosine kinase profiling
Source: Oncotarget. 2015 Aug 13;6(30):30057–71. doi: 10.18632/oncotarget.4996 (PMC4745781; doi:10.18632/oncotarget.4996)
Supplement: Supplementary file 1 [file oncotarget-06-30057-s001.pdf]

SUPPLEMENTARY FIGURES

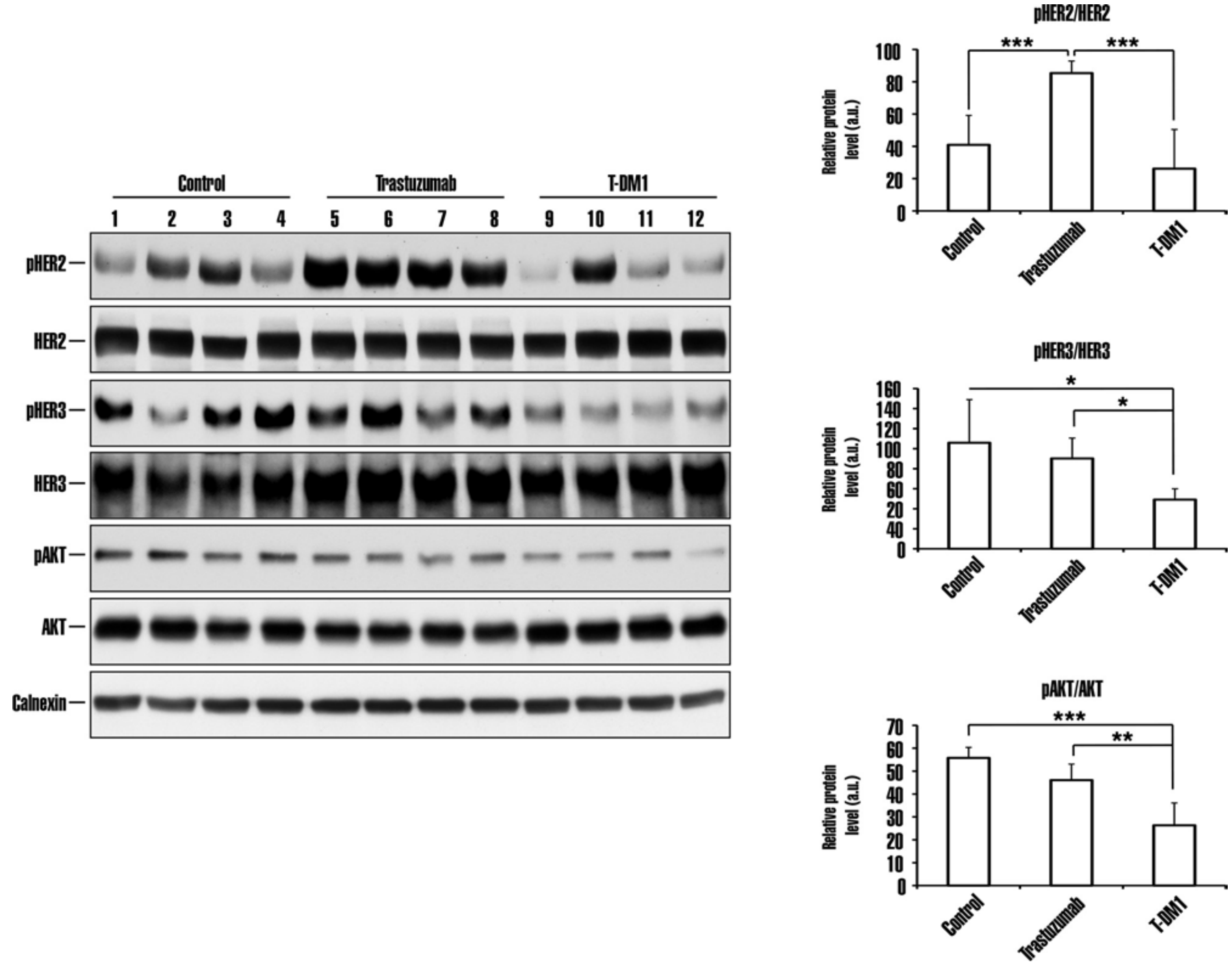

Supplementary Figure S1: Analyses of pHER2, pHER3, pAKT, HER2, HER3, and AKT levels in tumors from mice. SKOV3 cells were injected in mice and when tumors reached 500 mm^3, mice were randomized in three groups (control, trastuzumab, T-DM1, n = 3/group) and treated once per week (two treatments in total). Two days after the second treatment, tumors of different groups were obtained and immediately frozen in liquid nitrogen. The tumours were minced, washed with phosphate buffered saline and homogenized in ice-cold lysis buffer. Expression of pHER2, HER2, pHER3, HER3, pAKT, AKT and Calnexin were analyzed by Western blot. The graphs on the right represent the mean ± s.d. phosphorylation of pHER2, pHER3 and pAKT with respect to the total level of each protein in the different groups (Control, Trastuzumab and T-DM1). Student's t test were used to analyze differences among different groups. $*p < 0.05$, $**p < 0.01$, $***p < 0.005$.

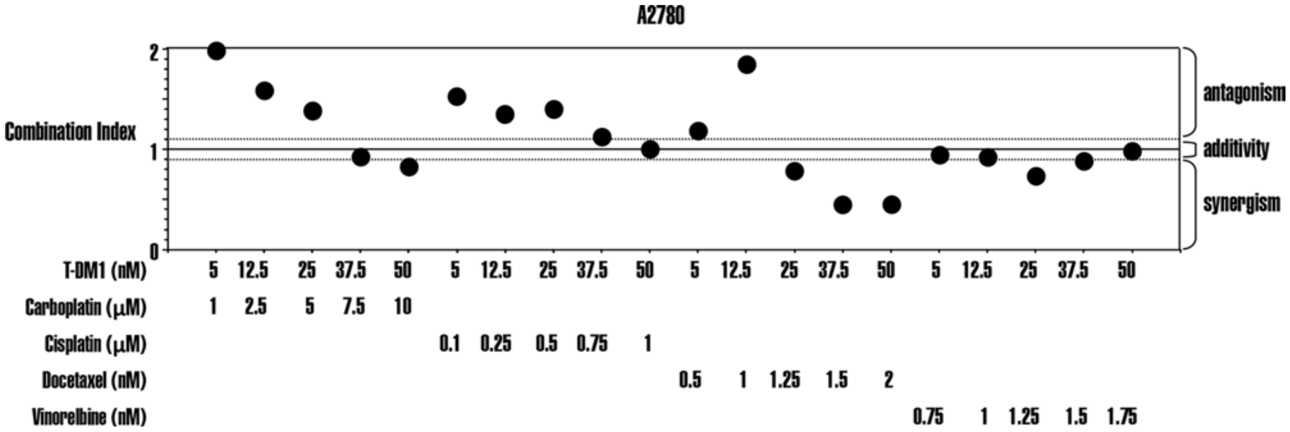

Supplementary Figure S2: Effect on A2780 of the combination of T-DM1 with chemotherapeutic agents used in ovarian cancer. A2780 cells were treated with the indicated doses of the drugs and their MTT metabolization were measured. Combination indexes for the different drug combinations were obtained using the CalcuSyn program and plotted.

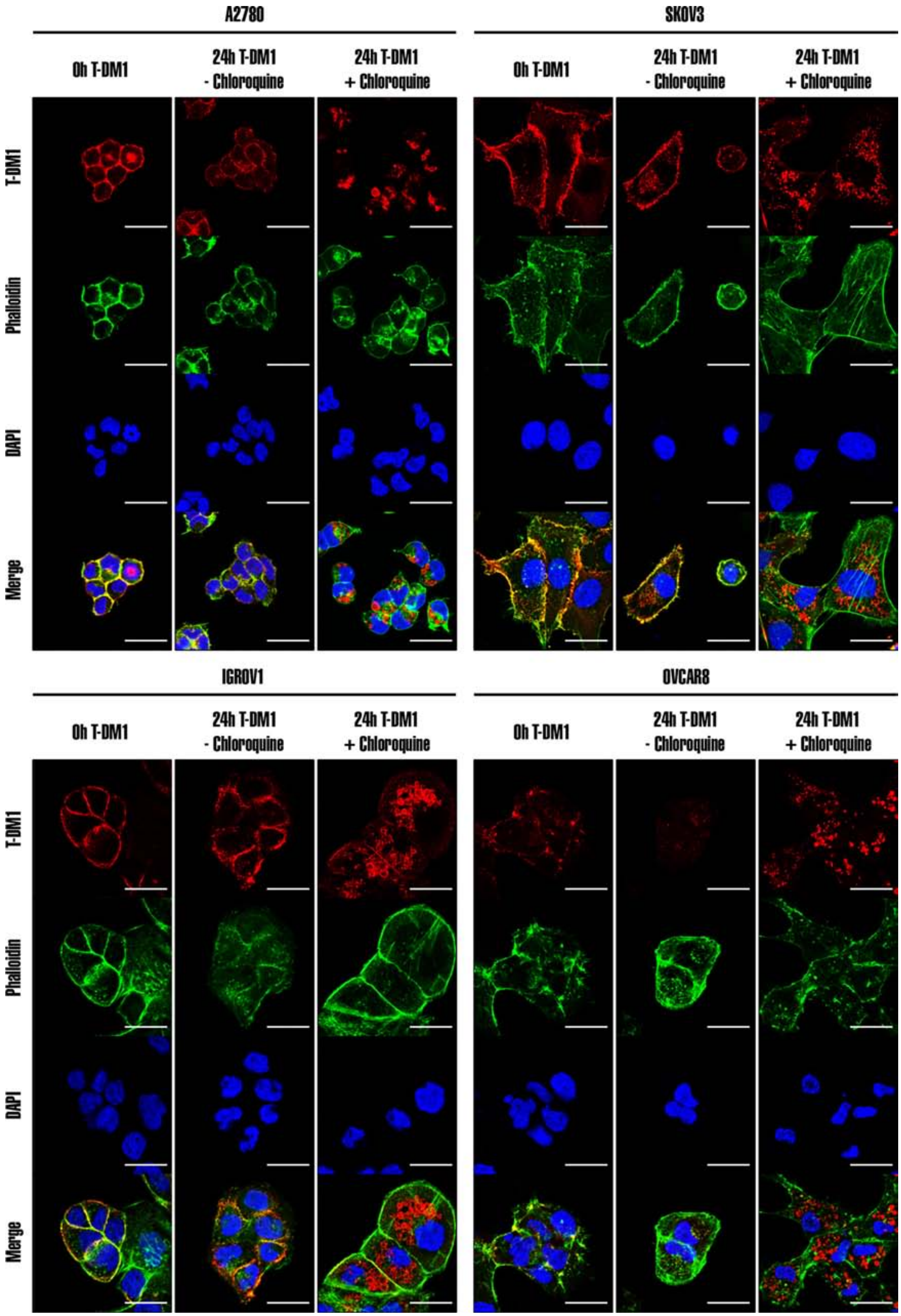

Supplementary Figure S3: Internalization of T-DM1 in four ovarian cancer cell lines. Cells were seeded on coverslips and treated with 50 nM T-DM1 for 2 hours. After washing out the drug, cells were cultured for 24 hours with or without 50 mM chloroquine. Cells were fixed and stained for T-DM1 (red), actin (green) and DNA (blue). Scale bar = 25 µm.
